# Smoking Health Professional Student: An Attitudinal Challenge for Health Promotion?

**DOI:** 10.3390/ijerph9072550

**Published:** 2012-07-23

**Authors:** Daniel Cauchi, Julian Mamo

**Affiliations:** Department of Public Health, Malta Medical School, Mater Dei Hospital, Birkirkara Bypass, Msida MSD 2090, Malta; Email: julian.mamo@um.edu.mt

**Keywords:** tobacco, smoking, prevalence, health profession

## Abstract

Tobacco is a major preventable cause of premature morbidity and mortality. Health professionals are uniquely positioned to provide targeted interventions and should be empowered to provide cessation counselling that influence patient smoking. A cross-sectional national survey was administered to all third year students in four disciplines at the University of Malta. The Global Health Professional Student Survey (GHPSS) questionnaire was distributed to collect standardised demographic, smoking prevalence, behavioural, and attitudinal data. 81.9% completed the questionnaire (n = 173/211). A positive significant association between tobacco smoke exposure at home and current smoking status was identified. Non-smokers regarded anti-tobacco policies more favourably than smokers, being more likely to agree with banning of tobacco sales to adolescents (OR 3.6; 95% CI: 2.5–5.3; *p* ≤ 0.001); and with a smoking ban in all public places (OR 8.9; 95% CI: 6.1–13.1; *p* ≤ 0.001). Non-smokers favoured a role for health professionals in promoting smoking cessation (OR 5.1; 95% CI: 3.1–8.5; *p* ≤ 0.001). Knowledge of antidepressants as tools for smoking cessation was also associated with a perceived role for skilled health professionals in cessation counselling (OR 4.9; 95% CI: 1.8–13.3; *p* = 0.002). Smoking negatively influences beliefs and attitudes of students toward tobacco control. There is a need to adopt a standard undergraduate curriculum containing comprehensive tobacco prevention and cessation training to improve their effectiveness as role models.

## 1. Introduction

Tobacco use is one of the major preventable causes of premature death and disease in the World. The World Health Organization (WHO) estimates that globally, over 1 billion people currently smoke tobacco [1]. It also attributes approximately 6 million deaths a year to tobacco, and this is expected to rise to around 10 million per year by 2030 [2]. 

Health professionals are uniquely positioned in that simple, targeted, brief and well-timed interventions on their patients can have an impact on smoking habits, particularly through effective patient counselling [3]. Health professional students equipped with knowledge to provide smoking cessation skills to future patients may also play a crucial role in reducing tobacco use and ultimately reducing smoking-related deaths [4,5]. There is a need for health care facilities and schools to assume a greater responsibility for students’ tobacco education by promoting non-smoking facilities and practices [6]. While physician smoking prevalence in developed countries seems to have decreased over the past decades, this is not the case for several southern European countries and developing countries [7,8], where an expanding body of evidence shows that the prevalence of tobacco smoking is rather high in current health professional students [9,10,11,12]. However, few studies have collected standardised information regarding future health professionals’ tobacco use, exposure to second-hand smoke, and training to provide cessation counselling. In fact, smoking issues are often taught in a non-systematic manner and are of limited quality in many countries [13,14,15]. 

The WHO and the U.S. Centre for Disease Control and Prevention, have attempted to overcome these limitations by developing and implementing the Global Health Professions Student Survey (GHPSS), which includes surveys of dental, medical, nursing, and pharmacy students’ tobacco habits. Results of the survey have led authors to advocate the introduction of a separate tobacco module in medical schools to counter these worrisome trends [10,16]. Malta is a small Mediterranean island which first participated in the GHPSS in 2010. In 2008 the prevalence of tobacco use of the Maltese adult population stood at 20.4% daily smokers and a further 5.5% as occasional smokers [17]. Very limited data on the smoking habits of Maltese health professionals/students had been collected previously, although a EUROPREV self-reported survey in 2000 indicated that 12.8% of Maltese GPs were regular smokers [18], and a small study carried out in 2009 on a representative sample of Maltese doctors and medical students (n = 71) indicated a smoking prevalence of around 5% [19]. The aim of this survey was to assess whether smoking habits influenced Maltese health professional students’ attitudes towards tobacco control.

## 2. Methods

### 2.1. Design

The Global Health Professionals Student Survey (GHPSS)—part of the Global Tobacco Surveillance System—is an international school-based cross sectional survey of third year students pursuing advanced degrees in dentistry, medicine, pharmacy, and nursing [16]. A self-administered, anonymous and validated [20] questionnaire was distributed to collect demographic, prevalence, behavioural and attitudinal data on tobacco use and cessation among health professional students. Fieldwork was carried out between January and May 2010 at the Malta Medical School and the Institute of Health Care. In Malta the medical, dental and pharmacy undergraduate courses last five years, whereas nursing students pursue one of two undergraduate courses of 3 and 4 years of duration, respectively. Malta is unique in that its small size and single acute general teaching hospital allow for a limited number of health care students across all disciplines to graduate each year. Thus, the sample population (n = 211) contained *all* third year students across all eligible disciplines in Malta, and the results can be used to make important inferences concerning tobacco use and risk behaviours of third year health professional students in the country. Ethical approval was obtained through the University of Malta Research Ethics Committee. The tool included questions on demographic characteristics, smoking habits, use of alternative tobacco products, attitudes and beliefs towards tobacco control activities and the educational training received with regards to smoking and smoking cessation. Prior to questionnaire distribution, all students were informed about the main objectives of the study and provided consent for their voluntary participation. 

### 2.2. Measurement

The study provided information on cigarette smoking; exposure to second-hand smoke (SHS) at home and in public places; and whether existing smoking ban policies were enforced. In addition, questions on attitude were asked to assess whether health professionals should be role models for their patients, whether training in smoking cessation techniques should be provided in undergraduate curricula and whether students had ever received formal training on such techniques. For the purposes of the study, ‘smokers’ were defined as those who had smoked cigarettes on one or more days during the previous 30 days, as several reviews suggest that even stable light or occasional smoking can carry substantial adverse health effects [21,22]. 

### 2.3. Statistical Analysis

Predictive Analysis SoftWare: PASW 18 (IBM Statistics) was utilized for statistical analysis, whilst weighted prevalence estimates were calculated by the Centre for Disease Control (Atlanta) using Survey Data Analysis: SUDAAN (RTI International) software. Descriptive results are presented as percentages. A finite population correction factor was applied to take into account non-response and used in the variance of the estimates. Univariate analysis was carried out using chi-square testing, with a *p* value of <0.05 taken as the threshold for statistical significance. All results have a margin of error of ±5% (95% confidence interval [CI]).

## 3. Results

### 3.1. Description of Participants and Smoking Status

The study population consisted of 211 students enrolled in their third year of study across the four undergraduate health professional schools. All disciplines and all health professional schools accepted to participate in the Maltese GHPSS. Student response rates are summarised in Table 1. Among the total population, 73.8% were female and 26.2% were male. Most of the respondents (93%) were aged between 19 and 24 years. 

**Table 1:** Health professional student response rate and smoking prevalence (95% CI).

Discipline	Dental	Medical	Nursing	Pharmacy	Total
Students (n)	7	77	86	41	211
Respondents	6	59	78	30	173
Response rate (%)	85.7	76.6	90.7	73.2	82.0
% of total sample population (n = 173)	3.5	34.1	45.1	17.3	100
% current smokers (95% CI)	60 (24.8–87.2)	14.3 (10.0–20.0)	40.3 (36.9–43.8)	13.3 (7.9–21.5)	/

### 3.2. Smoking Status

Of the 65.9% of all respondents who reported experimenting with cigarettes in the past (including one or two puffs), 27.9% were introduced to cigarettes at 11–15 years of age, and another 19% started experimented with smoking at 16–17 years of age. At the time of the survey, 123 (72.8%) were non-smokers, whereas 46 (27.1%) smoked either daily (n = 16, 9.5%) or occasionally (n = 30, 17.8%) during the previous 30 days. There was no significant difference in smoking status between genders (Odds Ratio (OR) 0.96; 95% CI: 0.45–2.1).

### 3.3. Tobacco Policy Awareness

A majority of students (88.7%) were aware of the existence of a non-smoking policy within the hospital grounds. Of these, 64.9% believed that the policy was enforced, while 24.4% disagreed with this view. Only 11.3% of all students surveyed were unaware of the school policy. There was no significant difference between the responses of smokers versus non-smokers with regards to knowledge of the non-smoking policy (*p* = 0.212) or perception of its enforcement (*p* = 0.226).

### 3.4. Home Environment and Smoking Status

Smokers were significantly more likely to report exposure to second hand smoke within their home (OR 3; 95% CI: 2.27–4.15; *p* ≤ 0.001) as well as outside the home environment, (OR 2.1; 95% CI: 1.3–3.2; *p* = 0.001) than were non-smokers.

### 3.5. Beliefs and Attitudes towards Smoking by Smoking Status

Table 2 summarises students’ attitudes toward tobacco control and smoking according to their smoking status. Although generally, all health professional students were against tobacco promotion irrespective of their own smoking status, non-smokers were more likely than smokers to say that tobacco sales to adolescents should be completely banned (OR 3.6; 95% CI: 2.5–5.3); and to agree with a smoking ban in discotheques, bars and pubs (OR 12.1; 95% CI: 8.4–17.4) in restaurants (OR 3.5; 95% CI: 2.1–5.7) as well as in all public places (OR 8.9; 95% CI: 6.1–13.1).

Odds Ratios calculated through regression analysis showed that in general, non-smokers were more positive than smokers in their attitudes towards regulation of tobacco sales, enforcement of tobacco-free zones and the perceived role of health professionals in reducing the burden of tobacco. With regards to the perceived role of health professionals in tobacco control and smoking cessation, most students (90%) believed health professionals should receive training on smoking cessation, with non-smokers being more evidently in favour (OR 3.5; 95% CI: 2.3–5.4; *p* ≤ 0.001). However, not all students seemed to believe in leading by example, as only 65% of all students think that health professionals should be role models, with no statistically significant difference between smokers and non-smokers (*p* = 0.1). Non-smokers were also more likely to believe that health professionals have a role in giving cessation advice routinely to patients (OR 5.1; 95% CI: 3.1–8.5; *p* ≤ 0.001), although they were only slightly more positive than smokers in their beliefs regarding the effectiveness of their advice (*p* = 0.079). Significantly, non-smokers were more likely to believe that patients had a reduced chance of being advised to quit by smoking health professionals (OR 3.5; 95% CI: 2.6–4.8; *p* ≤ 0.001). 

There was general agreement that students were taught about the harmful effects of smoking at undergraduate level, with non-smokers showing greater awareness (OR 1.7; 95% CI: 1.1–2.6; *p* = 0.02), however little attention seems to be given to the psychological basis of smoking, with around half the student population stating that they had not discussed reasons why people smoke (Table 3). 

Moreover, it was noted that smokers had a heightened awareness about the importance of taking an accurate tobacco history (*p* ≤ 0.001). The majority of students did not report receiving formal training in smoking cessation thus far in their curricula, and it is not known whether those who replied positively to this question (12.5%) received such training within the framework of their undergraduate studies. Additionally, while the majority had heard of nicotine replacement therapies, only a third of respondents were aware of antidepressant use in cessation programs, and less than two thirds of students had been taught that providing educational quitting materials is important.

The effect of formal tobacco control education on students’ beliefs about their role as models in providing smoking cessation advice was explored using logistic regression analysis after controlling for current smoking status (Table 4). Students reporting knowledge of antidepressants as tools for stopping smoking were more likely to report that health professionals have a role in smoking cessation (OR 4.9; 95% CI: 1.8–13.3; *p* = 0.002) and should receive cessation training (OR 2.2; 95% CI: 1.4–3.5; *p* = 0.001). A slight positive association was found between having knowledge of the adverse effects of smoking and belief that health professionals should routinely give cessation advice, however this association was not statistically significant (OR 2.2; 95% CI: 0.8–6.0; *p* = 0.123).

**Table 2:** Response to questions regarding attitudes towards tobacco control.

Respondents who answered yes to the question…	Total	Smokers	Non-smokers	*p* value
	% (n)	% (n)	% (n)	(2-sided)
Should tobacco sales to adolescents be banned?	85.2 (144)	71.7 (33)	90.2 (111)	**<0.001**
Should advertising be completely banned?	71.6 (121)	63.0 (29)	74.8 (92)	0.001
Do you agree with smoking ban in restaurants?	92.3 (156)	84.8 (39)	95.1 (117)	**<0.001**
Do you agree with smoking ban in discos/bars/pubs?	78.7 (133)	45.6 (21)	91.0 112)	**<0.001**
Do you think that smoking in all public spaces should be banned?	83.4 (141)	58.7 (27)	92.7 (114)	**<0.001**
Should health professionals get cessation training?	90.0 (152)	80.4 (37)	93.5 (115)	**0.012**
Are health professionals role models?	65.0 (110)	60.9 (28)	66.7 (82)	0.1
Should health professionals give quitting advice routinely?	92.2 (155)	82.2 (37)	96.0 (118)	**<0.001**
Should health professionals advise stopping other products?	89.9 (152)	82.6 (38)	92.7 (114)	**<0.001**
Do health professionals have a role in giving advice?	97.6 (165)	91.3 (42)	100 (123)	**<0.001**
Do chances of quitting improve if health professional gives advice?	79.8 (135)	76.1 (35)	81.3 (100)	0.079
Are health professionals who smoke less likely to advise patients to stop smoking?	68.6 (116)	47.8 (22)	76.4 (94)	**<0.001**

**Table 3:** Response to questions regarding tobacco education.

Respondents who answered yes to the question...	Total	Smokers	Non-smokers	*p* value
	% (n)	% (n)	% (n)	(2-sided)
During classes, were you taught about dangers of smoking?	88.7 (150)	84.7 (39)	90.2 (114)	**0.02**
Did you discuss reasons why people smoke?	52.1 (88)	56.5 (26)	50.4 (62)	0.10
Did you learn that it is important to record tobacco use history?	92.9 (157)	100 (46)	90.2 (111)	**<0.001**
Have you ever received formal training in smoking cessation?	12.6 (21)	11.1 (5)	13.1 (16)	0.42
Did you learn it is important to provide educational quitting materials?	62.1 (105)	65.2 (30)	61.0 (75)	0.24
Have you ever heard of nicotine replacement therapies?	95.9 (162)	95.7 (44)	95.9 (118)	0.85
Have you heard of antidepressant use in cessation programs?	33.9 (57)	42.2 (19)	30.9 (38)	**0.002**

**Table 4:** Adjusted odds ratio (OR) ^†^ and 95% confidence intervals (CI) for the role of education on health professional students’ beliefs towards smoking cessation and training.

	*Educational aspects*						
Beliefs	Knowledge of adverse effects of smoking	Reasons why people smoke	Importance of tobacco history	Importance of providing counselling material	Use of antidepressants in smoking cessation	Received formal training	Nicotine replacement
Health Professionals have a role in smoking cessation	**	0.8 (0.3–1.8)	**	0.5 (0.2–1.2)	**4.9** (1.8–13.3)	**	1 (0.02–0.3)
Health professionals should receive cessation training	1.1 (0.6–2.1)	1.3 (0.8–2.0)	**	0.6 (0.4–1.0)	**2.2** (1.4–3.5)	**	0.7 (0.3–1.6)
Health professionals are role models	1.2 (0.8–1.9)	1.2 (0.6–1.1)	1.6 (0.9–2.8)	1.0 (0.8–1.4)	1.3 (0.9–1.7)	0.9 (0.6–1.4)	**
Health professionals should give routine quitting advice	2.2 (0.8–6.0)	0.6 (0.4–1.0)	0.4 (0.2–1.1)	1 (0.6–1.7)	1.8 (1.1–3.0)	1.3 (0.6–2.7)	0.5 (0.2–1.2)
Do chances of quitting improve if a health professional gives quitting advice?	0.5 (0.3–0.8)	0.6 (0.4–0.8)	0.4 (0.2–0.7)	0.6 (0.4–0.8)	1.7 (1.2–2.4)	0.4 (0.2–0.7)	**

^†^ Adjusted for smoking status (smoker/non-smoker); ** not enough subjects to perform the analysis.

## 4. Discussion

### 4.1. Main Findings

Findings from the Malta GHPSS show that more than a quarter of health professional students are daily or occasional smokers; a rate slightly higher than that (19.3%) found in the corresponding adult Maltese population of the same age [17]. In this study, pharmacy students showed the lowest smoking prevalence. Medical students had a slightly higher prevalence, though this was still far higher than that found among medical doctors in a 2009 study [19].

Additionally, within the specific disciplines, the percentage of current medical student smokers was considerably lower than that of other European countries including Germany (28%), Italy (31.3%) and Poland (33.1%), but similar to that found in Spain (15.7%) [12]. However, smoking prevalence in nursing students was similar to Italy (48.2%) [23] but higher than that in most other participating European countries, including Greece (33.1%), Slovakia (32.2%) and the Czech Republic (32.7%) [11,24]. The number of dental students was considered to be too small to provide meaningful prevalence comparisons with other countries, whereas data on smoking prevalence in pharmacy students in other countries was generally limited. 

With respect to attitudes, the majority of students seem willing to ‘practice what they preach’, with most non-smokers having satisfactory attitudes towards tobacco control interventions and their potential role model status. On the other hand, among those health professional students that smoke, support for such efforts appears to weaken when these threaten their freedom to smoke in public places. This complements findings which suggest that students’ attitudes towards tobacco control may be linked to their established smoking pattern prior to starting their professional training, which in turn colours their perceptions of tobacco control [25]. 

Unlike findings from other studies [24] few significant links between previous tobacco education and students’ current attitudes towards tobacco control were elicited. This may be due to the general paucity of systematic training at undergraduate levels, or indeed due to a general lack of experience in practicing their acquired health promotional skills. 

Additionally, it is of concern that smokers seem dissatisfied with the national smoking ban in public places of entertainment—in place since April 2004, and unwilling to be assigned a role model status which might necessitate stopping smoking themselves. While the majority of smokers had positive attitudes towards policy efforts to control tobacco use, they were significantly less likely to agree to bans in public places when compared to non-smokers. This skepticism is also seen elsewhere [26,27]. Furthermore, smokers seemed to disagree with the idea that their smoking status might influence the advice given to patients, and are less willing to conduct health promotion specifically against tobacco.

Physician and nurse smoking status and tobacco control counselling is known to positively influence patient smoking habits [4,5] hence developing systematic tobacco cessation training; providing a smoke-free environment and encouraging medical and nursing students to quit is likely to play a significant role in the effectiveness and success of future patient counselling encounters [11,28,29,30,31]. 

The undergraduate health professional courses in Malta do not contain a specific module on tobacco use, but rather relied on separate lectures across the subjects that illustrate the ill effects of smoking on physical health. At the time of the survey, school administrators reported that no structured training in smoking cessation was provided to students in the four disciplines, although information on the health effects of smoking and the existence of various cessation methods was provided throughout the undergraduate courses. Knowledge of the latter was seen to impact students’ beliefs about the need for formal training, complementing their perceived role in smoking cessation counselling as future health professionals. In February 2012, the smoking policy at the Malta Medical School and the Institute of Health Care was revised, only permitting smoking strictly outside hospital and school grounds. Free quitting services information and smoking cessation classes are provided to students, qualified professionals and hospital staff who smoke. This is likely to positively contribute to reduction of smoking prevalence in health professional students [28].

### 4.2. Limitations

The GHPSS is subject to several limitations. Third year students may not have had substantial interaction with patients in a clinical setting, so that these results may not be extrapolated to practicing health professionals. Response rates fluctuated slightly across the different disciplines, and in the case of pharmacy and dental students the result was that no inference to that sub-group could be made given the small numbers involved. This is also reflected in the relatively wide CIs. For this reason also, no intra-disciplinary comparisons in smoking habits and attitudes towards tobacco control could be made. 

Although the disciplines represented in the GHPSS surveys tend to be on the front line in terms of dispensing cessation advice in Malta, other important health professionals, such as physiotherapists and psychologists, were excluded. In addition, although a reliability study carried out in Italy has determined satisfactory validity and internal consistency of the questionnaire [20], no systematic evaluation at an international level has yet been carried out for GHPSS. However, reliability studies for similar tobacco-related questions in the United States have indicated good test-retest results [32]. Finally, data were extracted from self-reported questionnaires, which do not always provide reliable information [33]. 

### 4.3. What This Study Adds

This study establishes the prevalence of smoking among health professionals in Malta today and adds to the evidence that current smoking status directly influences beliefs and attitudes towards tobacco control, despite apparently sufficient knowledge regarding the harmful health effects of smoking. 

While newly imposed stricter smoking policies within hospital and school grounds are a welcome step in the right direction to discourage tobacco use among all health professional students, this should be combined with formal training in cessation methods. This would reinforce positive attitudes towards tobacco control and increase the effectiveness of counselling skills, particularly since the students themselves seem to be keen on such systematic training. Studies have suggested that training students about the implementation of tobacco cessation techniques and regarding the clinical treatment of tobacco dependence would greatly contribute to a reduction in patient smoking rates [14,24].

The highly significant positive correlations of a current smoking habit with overall negative attitudes to societal efforts at tobacco control and smoking cessation among Maltese health professional students highlights the need for the adoption of a standard curriculum inclusive of comprehensive tobacco prevention and smoking cessation training for these students. This has been shown to facilitate long-term changes in attitudes and behaviours [34] and would empower them to actively quit smoking themselves, thus increasing the likelihood of performing interventions compared to untrained students [35]. 

Undergraduate curricula should ideally be revised to enable students to manage smoking dependency with a clear understanding of basic clinical and community-based cessation methods, as successfully done in the USA [36]. A system of regular assessment to prospectively capture health professional students’ changing trends in tobacco use and attitudes towards tobacco control would prove useful in determining the effectiveness of various smoking prevention approaches over time. It is possible that smoking students become smoking professionals who are less effective at tobacco prevention, although their attitudes might change as patient contact increases. Further research is recommended in this regard. Ultimately, investment in the quality of education of health professionals will improve the effectiveness of health professionals [37], reaping significant health benefits for both the professionals themselves and their patients.
